# Metabolism and Development during Conidial Germination in Response to a Carbon-Nitrogen-Rich Synthetic or a Natural Source of Nutrition in *Neurospora crassa*

**DOI:** 10.1128/mBio.00192-19

**Published:** 2019-03-26

**Authors:** Zheng Wang, Cristina Miguel-Rojas, Francesc Lopez-Giraldez, Oded Yarden, Frances Trail, Jeffrey P. Townsend

**Affiliations:** a Department of Biostatistics, Yale University, New Haven, Connecticut, USA; b Department of Ecology and Evolutionary Biology, Yale University, New Haven, Connecticut, USA; c Department of Plant Biology, Michigan State University, East Lansing, Michigan, USA; d Program in Computational Biology and Bioinformatics, Yale University, New Haven, Connecticut, USA; e Yale Center for Genome Analysis (YCGA) and Department of Genetics, Yale University, New Haven, Connecticut, USA; f Department of Plant Pathology and Microbiology, The R.H. Smith Faculty of Agriculture, Food and Environment, The Hebrew University of Jerusalem, Rehovot, Israel; g Department of Plant, Soil and Microbial Sciences, Michigan State University, East Lansing, Michigan, USA; University of California, Berkeley

**Keywords:** artificial medium, asexual development, asexual-sexual switch, conidiospore, filamentous fungi, germination, natural medium

## Abstract

Fungal spores germinate and undergo vegetative growth, leading to either asexual or sexual reproductive dispersal. Previous research has indicated that among developmental regulatory genes, expression is conserved across nutritional environments, whereas pathways for carbon and nitrogen metabolism appear highly responsive—perhaps to accommodate differential nutritive processing. To comprehensively investigate conidial germination and the adaptive life history decision-making underlying these two modes of reproduction, we profiled transcription of Neurospora crassa germinating on two media: synthetic Bird medium, designed to promote asexual reproduction; and a natural maple sap medium, on which both asexual reproduction and sexual reproduction manifest. A later start to germination but faster development was observed on synthetic medium. Metabolic genes exhibited altered expression in response to nutrients—at least 34% of the genes in the genome were significantly downregulated during the first two stages of conidial germination on synthetic medium. Knockouts of genes exhibiting differential expression across development altered germination and growth rates, as well as in one case causing abnormal germination. A consensus Bayesian network of these genes indicated especially tight integration of environmental sensing, asexual and sexual development, and nitrogen metabolism on a natural medium, suggesting that in natural environments, a more dynamic and tentative balance of asexual and sexual development may be typical of N. crassa colonies.

## Abstract (precis)

One of the most remarkable successes of life is its ability to flourish in response to temporally and spatially varying environments. Fungi occupy diverse ecosystems, and their sensitivity to these environmental changes often drives major fungal life history decisions, including the major switch from vegetative growth to asexual or sexual reproduction. Spore germination comprises the first and simplest stage of vegetative growth. We examined the dependence of this early life history on the nutritional environment using genome-wide transcriptomics. We demonstrated that for developmental regulatory genes, expression was generally conserved across nutritional environments, whereas metabolic gene expression was highly labile. The level of activation of developmental genes did depend on current nutrient conditions, as did the modularity of metabolic and developmental response network interactions. This knowledge is critical to the development of future technologies that could manipulate fungal growth for medical, agricultural, or industrial purposes.

## INTRODUCTION

Fungi exhibit a diversity of morphology and natural history characteristics and can be found in nearly every environment inhabited by living organisms. Their dispersal via spores—and, in some cases, via hyphal fragments—spawns new opportunities over long distances but also creates unexpected environmental challenges for the initial growth of individual fungi (1–4). Many ascomycetes can produce resistant meiotic spores (ascospores) via sexual reproduction and/or multiple bouts of large numbers of mitotic spores (conidia; 5) via asexual reproduction. Conidia usually lack the thick cell walls or dark pigments that provide resistance against radiation or drought conditions that are characteristic of ascospores. In the typical life cycle of ascomycete fungi, vegetative growth is an adaptive mechanism functioning to maintain asexual reproduction via rapid hyphal growth and production of conidia versus reproducing sexually via production of resistant meiotic spores that survive harsh changes in the environment. Insight into the mechanisms of responses to environmental signals in fungi requires illumination of the genetics and biology of conidial germination—especially illumination of the mechanisms of response that are active during early vegetative growth leading to asexual and/or sexual reproduction.

Neurospora crassa, a model filamentous fungus that flourishes in postfire environments, has long been studied to understand fungal biology and ecology (6, 7). Morphological development during asexual growth in N. crassa has been characterized mainly via gene-by-gene study of specific developmental stages, and genes have been identified that are responsive to external environmental as well as internal environmental factors during N. crassa growth (8–17). Recently, methods of computational annotation of metabolic pathways associated with the N. crassa genome have improved (8, 18–21), incorporating the extensive history of biochemical genetics performed on N. crassa metabolism.

Regulation in response to properties of the environment plays a key role in the fundamental life cycle fork governed by the classic autoregulatory asexual-sexual switch (9, 10). As a general rule for fungi, nitrogen starvation inhibits conidiation and induces sexual development, resulting in slow, robust dispersal, and carbon starvation leads to conidiation and sexual development (11, 12). Nitrogen starvation has long been known to induce or upregulate synthesis of “sexual development genes” (*sdv*), most of which are responsive to mating-type expression, suggesting that fixed nitrogen is one of the key environmental regulators in N. crassa sexual development (13). The effects of carbon starvation on induction of reproduction are also associated with the specific downregulation of expression of a large set of genes (termed “carbon catabolite repression”) (14). Abundant carbon and nitrogen, in contrast, promote asexual growth, resulting in rapid dispersal.

There have been few genetics-of-development studies that have investigated the early stages in conidial germination in N. crassa and none that compared the effects of carbon supply and nitrogen supply (15–17, 20, 22). Conidial germination is rapid and dramatic, constituting a suite of morphological changes that must represent a challenge to regulation in the face of the sparsity of nutrients within the environments that conidia of N. crassa often encounter. Most studies on conidial germination of N. crassa have used standard artificial media, such as Vogel’s medium (23) and Bird medium (24), which contain an abundance of carbon and nitrogen sources that repress sexual development. With results determined on artificial media available as a basis for comparison, it is becoming increasingly feasible to design experiments that illuminate fungal ecology (25). Conclusions based on analysis of cultures on artificial media need to be investigated with nutritional resources approximating the natural environments in which the fungus grows and for which metabolic and developmental pathways have been naturally selected. These natural environments likely contain low concentrations of simple carbohydrates and organic acids. Using artificial Bird medium (BM), which promotes asexual development, as well as a more natural medium, i.e., maple-sap medium (MSM), which supports both asexual and sexual reproduction, we investigated the synchronous metabolic and developmental processes that occur during the germination of N. crassa conidia.

## RESULTS

We collected and compared genome-wide gene expression data associated with fine-scaled morphological differentiation during conidial germination in N. crassa cultured on artificial BM and natural MSM. Developmental differences between conidial germination on BM and conidial germination of MSM were quantified, and differential regulation in gene expression was observed for genes affecting histones and genomic methylation, hyphal development, transcription factors (TFs), and responses to environmental signals. The expression profiles of these genes enabled reconstruction and comparison of regulatory networks and metabolic pathways relevant to development on BM and MSM. Expression profiling led to the identification of genes with knockout (KO) phenotypes relevant to conidial germination and demonstrated differential expression of predicted isoforms in N. crassa, including isoforms of key regulators in the asexual-sexual switch.

### Conidial germination on different media.

**FIG 1:**
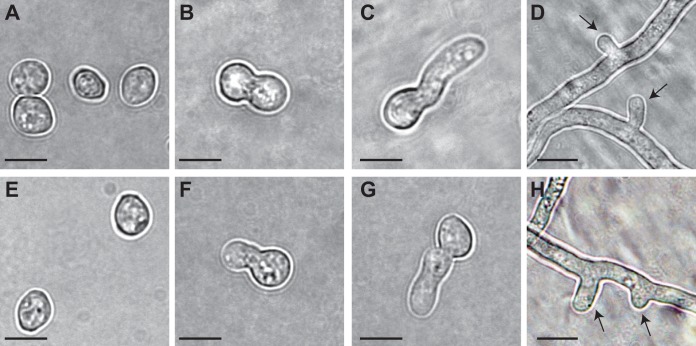
N. crassa asexual spores cultured on BM (A to D) and MSM (E to H) at four distinctive morphological stages of germination, corresponding to (A and E) fresh conidia, (B and F) polar growth, (C and G) doubling of the longest axis, and (D and H) the time of the first hyphal branching. Arrows indicate first hyphal branches. Scale bar, 5 μm.

**FIG 2:**
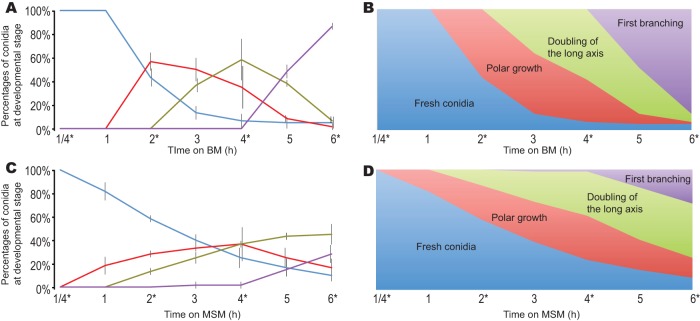
Temporal analysis of growth and development of conidia of N. crassa cultured on BM and MSM. Plated conidia were examined at six time points across the process, enabling sector counts that revealed the (A) proportions and (B) stacked proportions of conidia at serial stages of germination cultured on BM and the (C) proportions and (D) stacked proportions of conidia at serial stages of germination cultured on MSM. Measurements for conidia were color-coded at each stage of germination, including those corresponding to fresh conidia (blue), polar growth (red), doubling of the longest axis (green), and first hyphal branching (purple). An asterisk (*) indicates time points when RNAs were sampled. For staging, germination of 20 randomly selected conidia per plate was monitored; the error bars delineate 1 standard deviation of the mean for three such plates.

Nearly 50% of wild-type conidia germinated within 3 h after plating onto BM and MSM, which are similar in carbohydrate content but dissimilar in nitrogen and mineral content (Fig. 1; see also Table S1 in the supplemental material). Nitrogen content in MSM is too low to be detectable (Table S1), and it is known that low-nitrogen conditions promote sexual development in N. crassa (26). Germination commenced earlier on MSM (20% germination within the first 60 min) when compared to BM (almost no germination within the first 60 min). In contrast, extension of the germ tube and hyphal development were slower on MSM than on BM (Fig. 2). In plate tests, more conidia failed to germinate on MSM (6/60 = 10%) than on BM (3/60 = 5%), but the difference was not statistically significant (Fisher’s exact test, *P = *0.4906). Cultures on MSM started to produce protoperithecia and then perithecia within 10 days after inoculation and yet did so at a visibly lower density than is typically observed on synthetic crossing medium (SCM; 27), a low-nitrogen medium frequently used to induce sexual development in N. crassa (see Fig. S1 in the supplemental material).

10.1128/mBio.00192-19.1 FIG S1 Morphological development of N. crassa in (A to C) 3-day cultures and (D to F) 10-day cultures on (A and D) Bird medium (BM), (MSM) (B and E) maple sap medium, and (C and F) synthetic crossing medium (SCM). Abundant conidia were produced in the 3-day old cultures on (A) BM and (C) SCM. (D) No protoperithecia were detected in the 10-day cultures on BM. (E and F) Protoperithecia were produced in the 10-day cultures on MSM and SCM media (inset). Download FIG S1, PDF file, 2.3 MB. Copyright © 2019 Wang et al. 2019 Wang et al. This content is distributed under the terms of the Creative Commons Attribution 4.0 International license.

10.1128/mBio.00192-19.7 TABLE S1 The analytical compositions of BM and MSM. Download Table S1, DOCX file, 0.02 MB. Copyright © 2019 Wang et al. 2019 Wang et al. This content is distributed under the terms of the Creative Commons Attribution 4.0 International license.

### Sequencing mRNA during germination of conidia.

Culturing on both BM and MSM, we sampled RNAs from four conidial germination stages: fresh conidia, first polar growth of the germ tube, doubling of the germ tube length, and appearance of the first branch. A total of 40.6 to 94.5 million 76-bp paired-end reads were obtained from each sample (GEO accession no. GSE101412). Levels of total reads and mapped reads from RNA extracted from cultures on BM were slightly higher (6.8% and 7.3%, respectively, on average) than those from cultures on MSM (Table S2A). The average coverage depths were calculated following Illumina guidelines (*C* = *LN*/*G* [*C*, coverage; *G*, haploid genome length; *L*, read length; *N,* number of reads]), yielding 70× to 120× coverage, and the rate at which reads mapped to the genome ranged from 93.7% to 96.6% (Table S2A). The number of genes for which at least one read mapped to the gene for at least one time point on BM or MSM ranged from 9,167 to 9,202 (Table S2B and C).

10.1128/mBio.00192-19.8 TABLE S2 Analyses of RNAseq data. (A) Summary of transcriptome sequencing (RNA-seq) data and mapping quality. (B) Gene expression levels during conidial germination on BM. (C) Gene expression levels during conidial germination on MSM. Download Table S2, XLSX file, 3.9 MB. Copyright © 2019 Wang et al. 2019 Wang et al. This content is distributed under the terms of the Creative Commons Attribution 4.0 International license.

### Transcriptomics profiles during germination of conidia under different nutrient conditions.

Two major expression patterns—downregulation across all four stages and upregulation across stages after the second stage of germ tube appearance—can be recognized during conidial germination on BM. In contrast, expression patterns on MSM were more multifarious, with many genes upregulated from germination across stages until first branching event (Table S2B and C). Between the initial two stages (fresh conidia and polar growth), a large portion (1,818 genes) of the genome was significantly (Bonferroni adjusted *P < *0.01) downregulated, and these genes are associated with regulation of transcription, DNA binding RNA polymerase II transcription factor activities, and zinc ion binding (Table S2B and C). Significant functional enrichment (*P < *0.05) was identified for signaling pathways, cell cycle control, and carbon and nitrogen metabolism as well as for biosynthesis of amino acids both between BM and MSM cultures and across different morphological stages (Table S3A). Interestingly, mitogen-activated protein kinase (MAPK) signaling pathways were enriched at the last conidial germination stage but showed contrasting regulation patterns between BM and MSM.

10.1128/mBio.00192-19.9 TABLE S3 Functional analyses of selected genes during conidial germination in N. crassa. (A) Results of KEGG functional enrichment analysis for genes classified with stage-specific expression patterns. (B) Functional annotations of genes that were identified for the Bayesian network of asexual-sexual developmental responses to environmental factors during the conidial germination. Download Table S3, DOCX file, 0.05 MB. Copyright © 2019 Wang et al. 2019 Wang et al. This content is distributed under the terms of the Creative Commons Attribution 4.0 International license.

### Expression of genes in hyphal development characterized for conidial germination.

**FIG 3:**
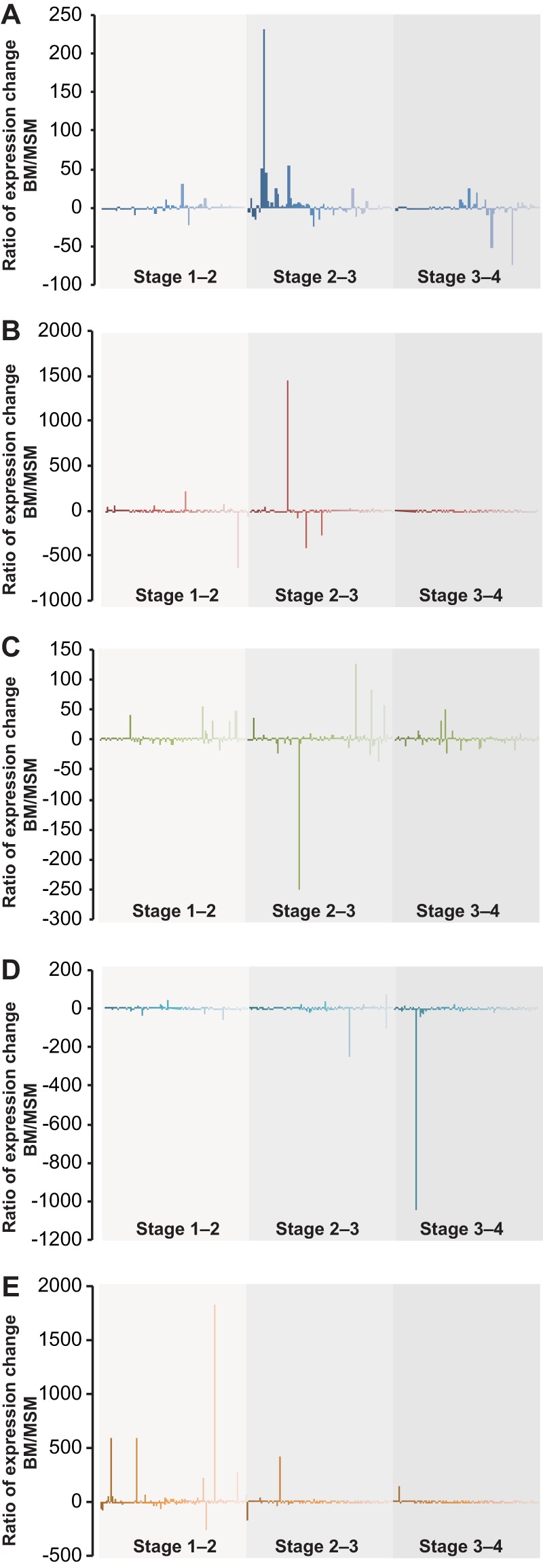
Effects of media on conidial germination are genome-wide, and functional groups differentially respond to BM and MSM during the process. Examples include (A) histone activities and genomic methylation, (B) hyphal development genes annotated as functioning in conidial germination, (C) transcription factors, (D) early light-responsive genes, and (E) late light-responsive genes. Genes within each panel are placed in a single order within each stage, with a diminishing color-shade corresponding to that order. Bars indicate ratios (BM/SM) of gene expression change from stage 1 to stage 2, stage 2 to stage 3, and stage 3 to stage 4.

Nutrition impacts during conidial germination were genome wide, affecting genes involved in development regulation (Fig. 3; see also Fig. S2 and S3). MAPK regulatory networks are known to regulate entry into serial morphological development stages as well as to activate the switch from the asexual phase to the sexual phase of the life cycle (28–31). For 59 genes that were able to be mapped to the MAPK signaling pathway of yeast (KEGG pathways) (32), expression exhibited much greater changes on MSM than on BM (Table S3; see also Table S4). Most (12/15) MAPK pathway genes expressed in response to starvation were significantly upregulated (*P < *0.01) on MSM (Fig. S2).

10.1128/mBio.00192-19.2 FIG S2 Differential expression of MAPK genes between BM and MSM cultures. Download FIG S2, PDF file, 0.1 MB. Copyright © 2019 Wang et al. 2019 Wang et al. This content is distributed under the terms of the Creative Commons Attribution 4.0 International license.

10.1128/mBio.00192-19.3 FIG S3 Differential expression of genes involved in hyphal growth between cultures on Bird medium and cultures on maple sap medium, including (A) genes involved in bud emergence and cell polarity, (B) genes involved in bud site selection, (C) genes involved in septins development and regulation, (D) genes involved in syntenic homolog actin-ring formation, (E) genes involved in syntenic homolog formation, (F) genes involved in transport and secretion during hyphal growth, (G) genes involved in morphological growth and hyphal polarity showing infrequent expression regulation in BM and MSM, and (H) genes involved in nitrogen metabolism showing differential expression regulation in BM and MSM. Download FIG S3, PDF file, 0.2 MB. Copyright © 2019 Wang et al. 2019 Wang et al. This content is distributed under the terms of the Creative Commons Attribution 4.0 International license.

10.1128/mBio.00192-19.10 TABLE S4 (A) Significant expression changes between stages observed for isoforms in Neurospora crassa during germination of conidia on BM and MSM. (B) Isoforms whose expression results were opposite and significantly different during germination of conidia on BM and on MSM. Download Table S4, XLSX file, 0.1 MB. Copyright © 2019 Wang et al. 2019 Wang et al. This content is distributed under the terms of the Creative Commons Attribution 4.0 International license.

**FIG 4:**
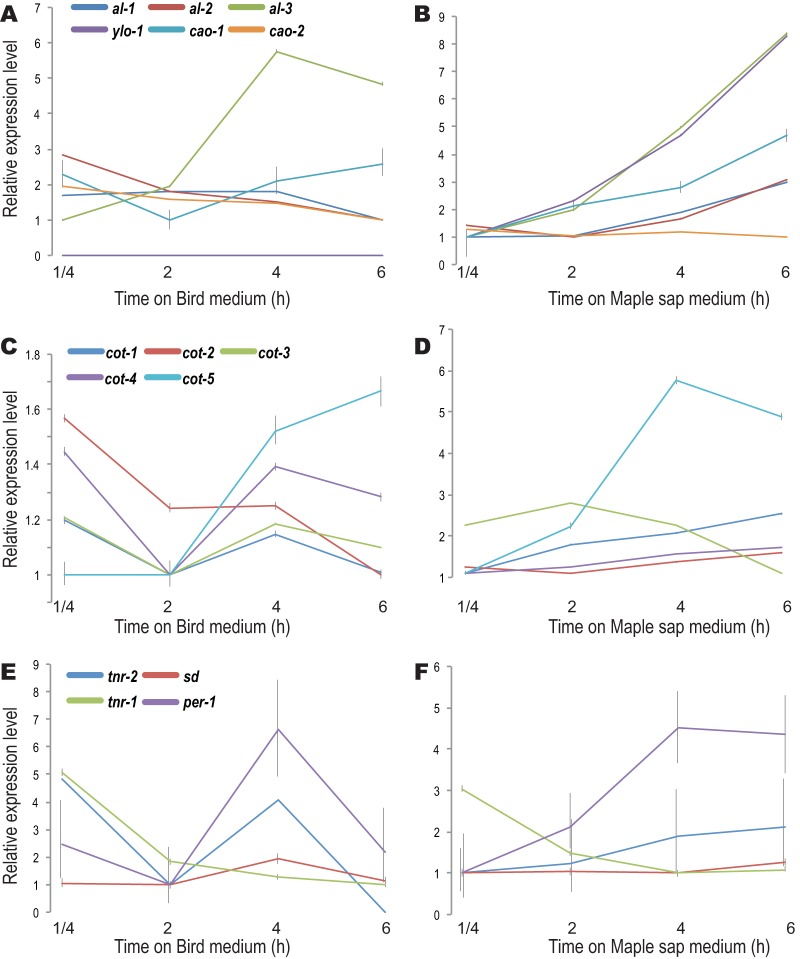
Relative expression levels of genes involved in carotenoid synthesis (pigmentation) pathways in culture on (A) BM and (B) MSM, of colonial temperature-sensitive asexual development genes (*Cot*) in culture on (C) BM and (D) MSM, and of a selection of genes whose expression is required for protoperithecial development in culture on (E) BM and (F) MSM.

Expression of genes associated with hyphal growth exhibited different patterns between BM and MSM (Fig. 4; see also Fig. S3). Genes associated with carotenoid synthesis were upregulated 2-fold to 7-fold on MSM but experienced nearly a 2-h delay in upregulation on BM (Fig. 4A and B). *cot* genes—which have been shown to confer colonial-temperature-sensitive (*cot*) phenotypes (33–37)—were first downregulated and then upregulated during germing tube extension on BM. These genes, excluding *cot-3,* were generally upregulated throughout the process for MSM cultures (Fig. 4C and D). Similar contrasts in expression levels between BM and MSM were also observed for genes related to polarity establishment, chitin synthesis, septation, and budding (Fig. S3A to E). Expression of genes required for sexual development was generally downregulated across stages on BM but was upregulated across stages on MSM (Fig. 4E and F).

### Expression of genes in metabolic pathways characterized for conidial germination.

**FIG 5:**
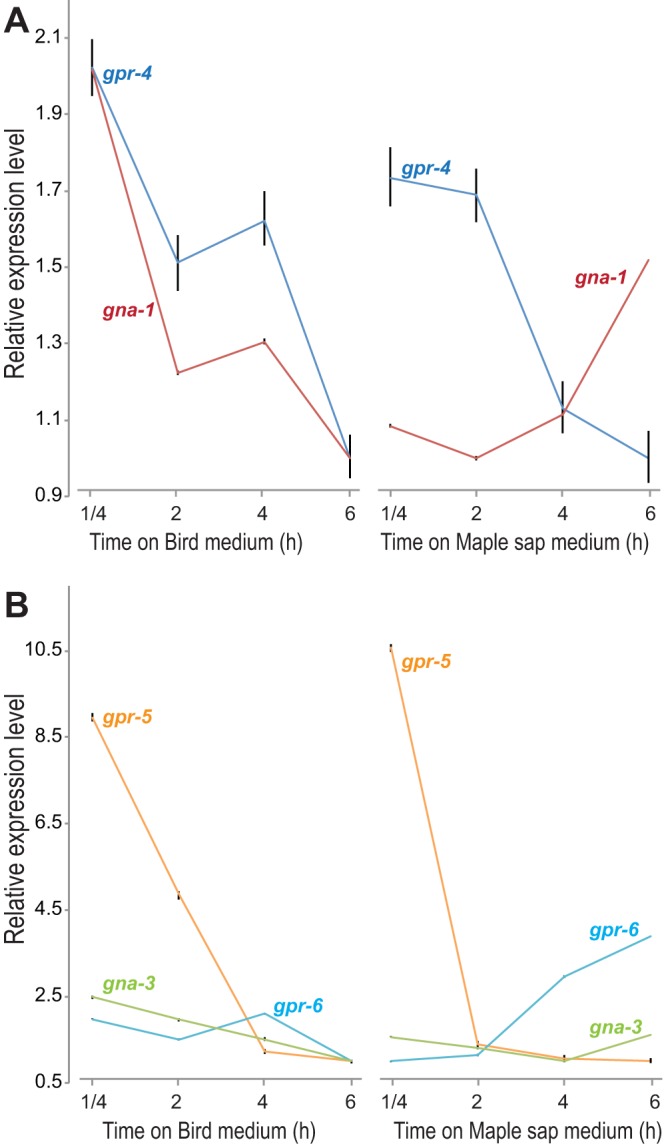
Relative expression levels of G-protein coding genes exhibited differential regulation during conidial germination on BM (left) and on MSM (right), including (A) cAMP signaling genes that regulate responses to carbon sources and (B) expression of genes, including genes *gpr-5* and *gpr-6* and corresponding potential coupled G-protein coding gene *gna-3*, that regulate responses to nitrogen starvation.

G-proteins and coupled receptors (*gpr*)—which are known to be critical regulators of fungal responses to carbon and nitrogen nutrition and major regulators of N. crassa development (38–41)—were differentially expressed. Genes *gna-1* and *gpr-4*—which regulate the responses to carbon sources (42)—were highly coordinately downregulated in cultures on BM but showed contrasting results in cultures on MSM, upon which *gna-1* was upregulated (Fig. 5A). Expression levels of *gpr-5* and *gpr-6* and the corresponding potentially coupled gene *gna-3* were highly coordinately downregulated on nitrogen-rich BM (Fig. 5B). Upregulated expression of *gpr-6* and *gna-3* in MSM with extremely low levels of nitrogen invites further investigation of their roles as potential nitrogen sensors in N. crassa.

Both nitrogen resources and carbon resources are critical for hyphal growth. Consistent with our observation of morphological development differences between BM cultures and MSM cultures, genes regulating polar growth of hyphae exhibited homogeneous regulation under both growth conditions (Fig. S3G). However, the nature of nitrogen metabolism regulation was complex. On nitrogen-deficient MSM, 14 of 17 genes for which homologs are annotated in yeast during nitrogen metabolism (KEGG pathways) were dramatically upregulated, including 51-fold, 81-fold, and 147-fold increases for nitrate transporter-10 (*nit-10*), NCU02361 (formamidase), and *nit-6*, respectively (Fig. S3H). As sucrose is the dominant carbon source in MSM, we observed stably high expression of the invertase gene (*inv*; NCU04265) during the first two stages of conidial germination on MSM but abrupt downregulation in expression of this gene on BM.

### Expression of transcription factors in conidial germination.

Two expression patterns were commonly observed for TFs in culture on BM; 48 TFs were steadily downregulated, exhibiting lowest expression at the last stage of development, and 36 exhibited their lowest expression at the second stage (polar growth). Expression of these TFs showed various expression patterns for MSM cultures. The TFs regulating both basal hyphal growth and asexual development showed similar downregulated expression patterns in RNAs obtained from cultures grown under both nutrient conditions. The exceptional transcription factors were *tah-1* (tall aerial hyphae) and *tah-4*, which were both significantly upregulated in both BM and MSM cultures. Among the 100 TFs profiled (Table S2B and C), some regulate both asexual development and sexual development (30, 43–45). Expression of *sub-1*, a key regulator in the asexual-sexual switch (45, 46), exhibited contrasting patterns of regulation between nutrient conditions: significant upregulation across stages on sexual development inducing MSM and significant downregulation across stages on nitrogen-rich BM.

### Expression of genes in response to environmental signals during conidial germination.

Many genes that exhibit early light-regulated responses (ELRGs [47]) were upregulated from germination to the first hyphal branching on MSM but were downregulated during the same stages on BM (Fig. S4A). For example, NCU01258 (*cyn-1*) encodes cyanate lyase in nitrogen metabolism and exhibited contrasting expression patterns between the cultures on nitrogen-rich BM and cultures on nitrogen-poor MSM. Upregulation of *cyn-1* indicates that the fungus had turned to complex compounds such as cyanate as an alternative nitrogen supply in MSM. Late light-regulated genes (LLRGs [47]) were generally upregulated during germ tube extension and hyphal growth (Fig. S4B). However, there was a dramatic downregulation of these genes before the appearance of germ tubes in conidia germinated on BM, including a nearly 70-fold drop for the *inv* gene (invertase; NCU04265), which catalyzes degradation of sucrose to glucose.

10.1128/mBio.00192-19.4 FIG S4 (A) Expression of early light-responsive genes (ELRGs) was generally downregulated in cultures on BM and MSM during the first two stages of conidial germination. (B) Expression of late light-responsive genes (LLRGs) was generally downregulated in cultures on BM but upregulated in cultures on MSM. Download FIG S4, PDF file, 0.1 MB. Copyright © 2019 Wang et al. 2019 Wang et al. This content is distributed under the terms of the Creative Commons Attribution 4.0 International license.

### Coordinated metabolic and developmental networks during conidial germination.

**FIG 6:**
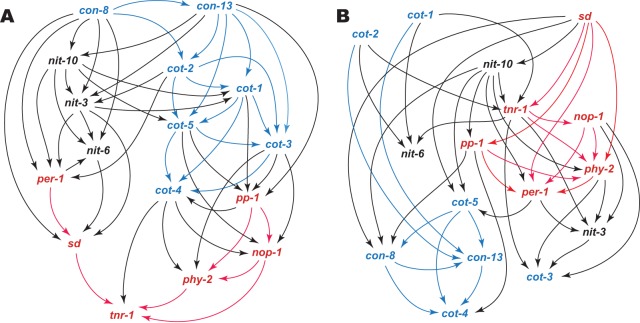
Bayesian networks of expression regulation show associations (corresponding to arrows indicating directed edges) among genes involved in nitrogen metabolism (black), asexual growth and reproduction (blue), and light-regulated sexual development (red) during conidial germination and early hyphal growth (A) on BM and (B) on MSM.

Key genes annotated in nitrogen metabolism, conidial germination, and the asexual-sexual switch were investigated for their associations in Bayesian coexpression networks (Fig. 6; see also Table S3B). Associations among genes associated with asexual development, including conidiation genes *con-8* and *con-13* and genes *cot-2*, *cot-1*, and *cot-5* regulating asexual growth and development, were conserved between cultures on BM and MSM. Associations among genes playing roles in the initiation of sexual development, including light sensor genes *nop-1* and *phy-2* as well as genes *per-1*, *pp-1*, *sd*, and *tnr-1*, were also conserved between the two cultural conditions. For cultures on nitrogen-rich BM, however, the asexual development subnetwork (*cot* genes) and nitrogen transport subnetwork (*nit* genes) were tightly modular. For cultures grown on nitrogen-poor MSM, which facilitates entry into both asexual development and sexual development, the asexual and sexual initiation modules were distinct but appeared less hierarchically organized, and the nitrogen metabolism genes were integrated with the developmental pathways much more extensively than with each other. For cultures on BM, the sexual development initiation subnetwork was positioned downstream, perhaps being responsive to expression regulation within the asexual development subnetwork—specifically, with respect to nitrogen metabolism. For cultures on MSM, the sexual development initiation subnetwork was located further upstream and was more integral to nitrogen transporter expression than on BM.

### Alternative splicing.

By analysis of our paired-end sequencing data, we differentiated among the expression results determined for isoforms of 898 predominantly metabolic genes that have been annotated with at least two isoforms (http://genome.jgi.doe.gov/Neucr2/) (Table S4A). Isoforms of 21 genes exhibited differential expression patterns between BM and MSM cultures, especially during early germination from stage 1 to stage 2 (Table S4B). Among the 17 isoforms that exhibited a significant (*P < *0.01) expression change for cultures on both media, isoforms of essential sexual reproduction gene *sub-3* (submerged protoperithecia-3; NCU01154T1) exhibited 2-fold downregulation on BM but 2.5-fold upregulation on MSM.

### Knockout phenotypes during conidial germination.

**FIG 7:**
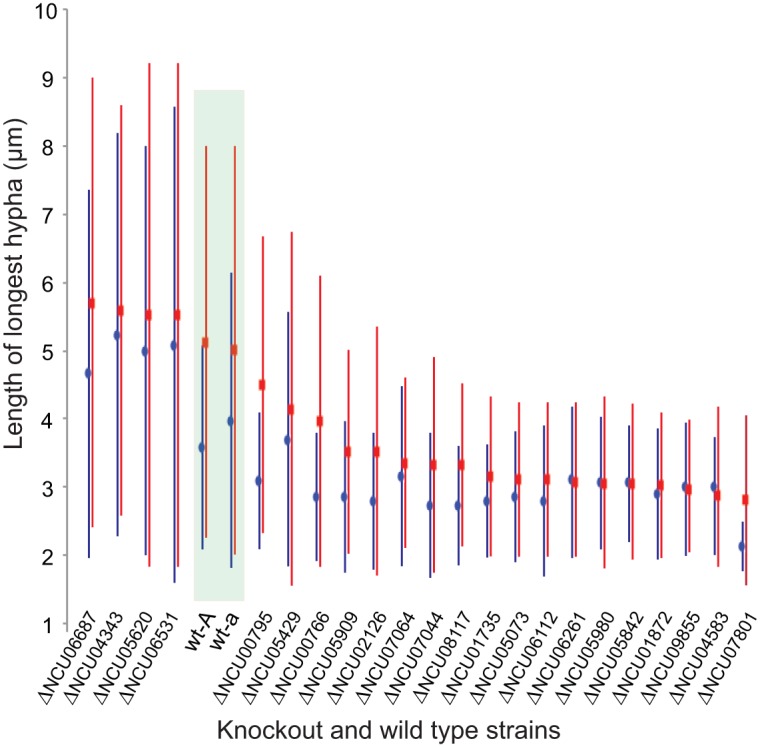
Knockout strains exhibited higher and lower average growth levels at 2 h (blue) and 3 h (red) postinoculation compared with wild-type strains of both A and a mating types (*wt-A* and *wt-a*). Phenotyping was performed on paired knockout and wild-type strains. Measurements of growth were conducted for three replicates of 20 randomly picked conidia for each strain. A high standard deviation value is evident, arising primarily as a consequence of a high variance in the starting time of germination.

**FIG 8:**
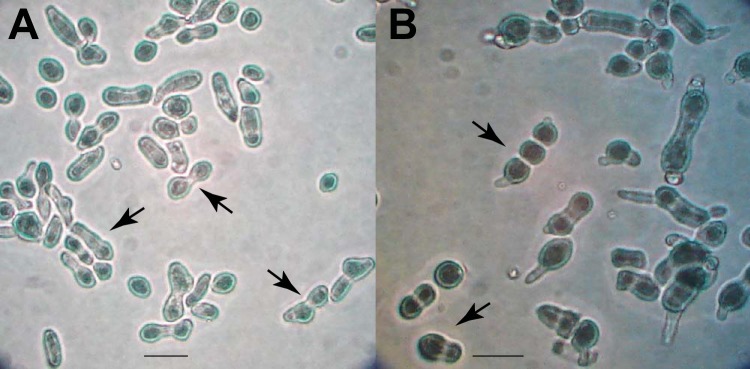
Phenotypes of knockouts of NCU08095 on BM at the stages of (A) polar growth and (B) doubling of the longest axis, exhibiting dumbbell-like conidial extension (arrows) before formation of the germ tube. Images of controls (representing wild-type germination on each medium) are shown in Fig. 1.

A total of 195 genes exhibited statistically (*P < *0.05) and biologically (>5-fold) significant differences between the two media in comparisons of data obtained from similar time points or across the conidial germination process. Among these genes, 144 knockout strains (43) were available from the Fungal Genetic Stock Center (FGSC [48]) for phenotypic investigation. In comparisons of wild-type strains with matched mating-type gene knockouts, we observed altered phenotypes in 22 of the knockout strains (Fig. 7; see also Fig. S5). Of the 22, only 13 genes are functionally annotated (Table 1). Generally, upregulation of expression was consistent with the time of function. For example, expression of a hypothetical protein was detected only in the early stages of germination, and the knockout of the corresponding gene—NCU07801 (*idler*)—resulted in significantly delayed germination and slower hyphal growth. In another example, one knockout exhibited a novel phenotype of spore elongation, in which conidiophores extended their long axis to form a dumbbell-like structure (NCU08095; *cdg* [conidia dumbbell germination]) before forming a normal germ tube (Fig. 8). Interestingly, expression of NCU08095 was significantly downregulated in both BM cultures and MSM cultures. Orthologs of *cdg* were found only in some genomes of Sordariomycetes, Leotiomycetes, and, Eurotiomycetes and were absent in other ascomycetes and yeast genomes—and the ortholog in *Rutstroemia* was annotated as a kynurenine formamidase (Fig. S6).

**TABLE 1:** Functional annotation for 13 genes that exhibited knockout phenotypes in conidial germination from previous studies^*a*^

Gene ID	Functional annotation at NCBI and FungiDB
NCU00795	Cation transporter
NCU02126	Isovaleryl-CoA dehydrogenase
NCU04343	Ergothioneine-1
NCU04583	Acetyltransferase
NCU05429	Alpha-glucan branching enzyme
NCU05620	Proteasome activator
NCU05980	Carboxypeptidase S1
NCU06112	Glutamate decarboxylase
NCU06261	Uracil phosphoribosyltransferase
NCU06687	Lycogen synthase-1
NCU07044	Metallo-beta-lactamase
NCU07064	l-Galactonate dehydratase
NCU09855	Nicotianamine synthase

a ID, identifier; FungiDB, The Fungal and Oomycete Genomics Resource (81); Isovaleryl-CoA, isovaleryl-coenzyme A.

10.1128/mBio.00192-19.5 FIG S5 Differential expression of genes that exhibited knockout phenotypes during conidial germination. Download FIG S5, PDF file, 0.1 MB. Copyright © 2019 Wang et al. 2019 Wang et al. This content is distributed under the terms of the Creative Commons Attribution 4.0 International license.

10.1128/mBio.00192-19.6 FIG S6 Phylogeny of orthologs of NCU08095, illustrating their presence or absence in genomes of Leotiomycetes, Sordariomycetes, and Eurotiomycetes. Branches with significant support (MrBayes posterior probability value, >0.95) are indicated in bold characters. Download FIG S6, PDF file, 0.1 MB. Copyright © 2019 Wang et al. 2019 Wang et al. This content is distributed under the terms of the Creative Commons Attribution 4.0 International license.

## DISCUSSION

In many cases, conidial germination is the critical first step of fungal colonization. We previously reported signatures of gene expression associated with mating type identifiable during asexual reproduction of N. crassa on BM (49). In this study, we investigated the conidial germination process and its responses to different nutritional conditions by profiling transcriptomics across four distinct morphological stages of conidial germination on two different media. Commercial maple sap provides a readily available natural nutrient source enabling investigation of diverse stages of N. crassa development, including conidial germination.

### Fungal spores prepare for flexibility instead of efficiency.

Nutrients represent a major factor influencing the onset of N. crassa colonization, and extended maintenance on a singular formulation of artificial medium is known to lead to degeneration of strains (50–52). Natural environments of N. crassa can be very diverse in terms of carbon level and nitrogen level, and this fungus has often been found to flourish in high-carbon environments, in particular, in the noncharred, sappy, woody remains of plants after forest fires. N. crassa has also been suggested to propagate as a plant endophyte (53); MSM resembles the nutrition accessible to the fungus within the environment of the living plant. While epigenetic regulation based on parent-of-origin expression from fungal species has been reported previously (54–56), our data exhibited no impact of parental nutrient conditions on conidial germination. Although the tested wild-type strains had been maintained on BM for multiple generations, the delayed germination of their conidiospores on BM is evidence that the conidia are equipped with germination surveillance that has evolved calibration toward a more natural environmental setting.

### Genetic associations between development and the metabolism of nutrition.

Our observations are consistent with previous research on the impact of levels of fixed nitrogen on sexual development and further suggest that these effects begin in the very early stages of asexual growth, during conidial germination. Nitrogen, in the form of nitrate, is the primary source of nitrogen nutrition and is often a limiting resource in natural environments (57). Therefore, fungal growth and development on a low-nitrogen natural medium likely represent a good model for most fungal colonization of natural environments. *S*everal standard media have been developed for studying N. crassa (58). Natural media such as carrot medium (59) have also been widely used in fungal research, especially to study pathogens whose growth conditions are challenging to mimic with artificial media (60, 61). While these media are useful for investigating specific stages in *Neurospora* growth and development, they all have limitations with respect to the study of growth stages that require quick changes in nutrients under laboratory conditions. BM and MSM provide different nutrition profiles (see Table S1 in the supplemental material), especially in nitrogen levels and carbon resources. MSM represents one of the likely environments for *Neurospora*, which has been reported to sometimes persist as a plant endophyte (53). Low nitrogen levels in MSM induced the initiation of sexual development even at very early stages of conidial germination, and key regulatory genes in sexual development were activated in MSM cultures (Fig. 3 and 4; see also Fig. S1, S2, and S4). Our results also call for further investigation of the involvement of G-proteins in nitrogen metabolism regulation in N. crassa. Upregulation of *gna-1* on MSM is consistent with previous suggestions indicating an additional function of *gna-1* in sexual development (62). Expression of *gna-1* was downregulated in cultures on BM, where sexual development is repressed. Gpr-5 and Gpr-6 are homologs of Stem1, a transmembrane protein in yeast that signals upon nitrogen starvation (6, 38, 63). Their roles as nitrogen sensors in N. crassa growth and development have not yet been confirmed (64).

Although expression was dynamically regulated in response to available carbon and nitrogen levels in both BM and MSM, the spores appear to take longer to physiologically adapt to BM than to MSM. To illustrate the metabolic responses, consider the expression of *cyn-1* (encoding cyanate lyase, a key enzyme in transforming cyanate as an alternative nitrogen resource), which was upregulated in cultures in nitrogen-poor MSM. Expression of invertase (encoded by *inv*)—which catalyzes degradation of sucrose to glucose—was, conversely, downregulated in glucose-rich BM (Table S2). In fact, two frequent expression patterns were identified on BM: continuous stage-to-stage upregulation and downregulated expression during germ tube formation followed by upregulated expression in subsequent stages. In contrast to these relatively homogeneous expression patterns, which are attributable to the presence of a high number of genes in BM cultures, expression patterns identified in MSM cultures were more multifarious, indicating more intricacy of function and developmental dynamism.

### Response of conidial germination to various environmental factors.

We observed greater upregulation of early light-induced genes (ELIG) in cultures on MSM than on BM. ELIG are controlled by the light-activated white collar complex (WCC) and regulate activities of late light-induced genes (LLIG) in growth, conidiation, and sexual development in N. crassa (47, 65–68). The upregulation of ELIG in MSM cultures is likely associated with boosted sexual development of N. crassa on MSM. Knocking out early light-induced gene NCU01870 yields a female sterile phenotype (68). This gene is expressed in a steady stage-to-stage increase in MSM cultures but is significantly downregulated stage to stage on BM, on which sexual development is inhibited. Using MSM helped us to reveal different aspects of gene regulation during the asexual-sexual switch by light-responsive WCC and metabolic pathways, which have been obscured on nitrogen-rich artificial media such as BM.

Our analysis illustrates that with respect to the adaptively tuned decision to engage in asexual or sexual development, metabolic pathways, sensory responses to environmental stimuli, and developmental pathways are tightly associated even early during conidial germination. The asexual-to-sexual switch is an integrative process, linking the asexual and sexual modes of reproduction—modes that respond to nearly opposite environmental attributes (11, 49). In many ascomycetes, asexual reproduction is prolonged by high temperatures and by high levels of nutrition, oxygen, ROS (reactive oxygen species), and light exposure, and sexual reproduction can be induced by low temperature, low levels of nutrition and oxygen, and reduced light intensity (69).

Although our experiment profiled expression only during the very early stages of conidial germination and hyphal extension, genes modulating the asexual-sexual switch showed the presence of regulatory networks that diverged between the two culture conditions. Bayesian networks based on coexpression illustrated that both asexual reproduction regulation and sexual reproduction regulation were highly modular in both BM and MSM cultures. However, nitrate transporters were modular in the expression network inferred from BM cultures, in line with intensive activation of nitrogen intake and metabolism. Multiple interactions between nitrogen metabolism and a similarly modular conidiation pathway suggest coordinated responses of nitrogen metabolism and conidiation to high-nitrogen media such as BM that are associated with the promotion of asexual growth and inhibition of sexual development. The more diffuse organization of these gene interactions could be a consequence or a cause of the more labile balance of the asexual-sexual switch in MSM.

### Complex genetic regulation of conidial germination implied by KO phenotypes.

Genes with critical roles in conidial germination were identified on the basis of comparative transcriptomics and focused knockout phenotyping. Of 23 genes identified for knockout phenotyping, 22 encoded knockout mutants that showed quantitative phenotypes with respect to germination ratio and growth rate. Knocking out NCU08095 (*cdg*) yielded a germination phenotype resembling yeast budding morphology—and this gene is not present in yeast genomes. Morphological transitions from filamentous growth to yeast-form growth have occurred many times in fungal evolution (70), and such physiological transformations are known to be associated with pathogenesis for some fungal species (71, 72).

Interestingly, no knockout strains showed distinct phenotypes on different media, despite differential expression on BM and MSM. One explanation for this homogeneity of knockout phenotypes across environments is that expression differences are largely associated with differential developmental timing on BM and MSM rather than being attributable to operatively different expression programs. In other words, most genes that we chose based on their expression differences between the cultures on different media likely represent quantitative regulators of developmental change, leading to quantitative differences in development pace, rather than qualitative differences in development, between cultures on BM and MSM.

Our results call for greater attention to isoforms of genes and their distinct functions during fungal development. For example, isoforms of the gene *sub-3*, which is essential for sexual reproduction, exhibited 2-fold downregulation when cultured on BM. In contrast, isoforms of *sub-3* exhibited 2.5-fold upregulation when cultured on MSM, on which sexual development can be expected to occur within a week after inoculation.

### Conclusion.

Synchronous metabolic and developmental processes underlying conidial germination, a rapid process that occurs in response to environmental signals, including carbon and nitrogen nutrition as well as light signals, were revealed by transcriptomics analyses performed with synthetic or natural nutrition. Results indicating modularity among elements of early sexual development, asexual growth, and nitrogen metabolism were detected in conidial germination, with a more diffuse set of network interactions in natural medium than in nitrogen-rich laboratory medium. The implication is that a more tentative balance of asexual and sexual development is typical during growth and development of N. crassa colonies in natural environments than has been previously implied by analyses relying on culture in media that suppress activation of the asexual-sexual switch. Nine genes that were previously unannotated with respect to function and that we have now identified as contributing to asexual growth after conidial germination may contribute significantly to modulating this balance and provide targets for future fungal growth control in prevention of pathogen infection, in biochemical fermentation optimization, and in bioenergy generation.

## MATERIALS AND METHODS

### Strains and culture conditions.

Germination studies were performed with N. crassa *mat A* (FGSC2489) macroconidia, harvested from 5-day cultures on solid (2% agar) Bird medium (24). Macroconidia were collected with deionized distilled water containing Tween 20 (0.1%). They were washed with autoclaved distilled water and filtered through a three-layer Mira cloth. Spores (1 × 10^5^) were placed on top of cellophane-covered medium in petri dishes. MSM was composed of maple sap (Vertical; Feronia Forests) with agar (2%). Conidia were incubated on media at 25°C under constant white light, a protocol that avoids the dynamic expression regulation known to arise from changes of light color and intensity (47). Germination was monitored at 0, 15, 60, 120, 180, 240, 300, and 360 min. Cellophane membranes with fungal tissues were collected at 15, 120, 240, and 360 min, when the majority (51% to 92%) of active spores on BM were at one of the following stages and beyond: fresh spores, spores showing evidence of polar growth, spores having doubled their long axis, and spores having commenced their first hyphal branching. The same time points were used for sampling tissues on MSM. Tissue samples were flash frozen in liquid nitrogen and stored at −80°C. All tissues that were collected from multiple plates in one collection process were counted as one biological replicate. Three temporally segregated biological replicates were prepared for each sampled time point on both BM and MSM.

### RNA isolation and preparation.

Total RNA was extracted from homogenized tissue with TRI reagent (Molecular Research Center) as described previously by Clark et al. (73). Preparation of cDNA used N_6_ primers following the Illumina mRNA sequencing sample preparation guide. The quality of the cDNA samples was verified with an Agilent 423 Technologies Bioanalyzer to ensure an insertion size of between 150 to 225 bp and by quantitative PCR (qPCR) (Kapa Biosystems) to ensure an RNA concentration of ≥0.5 ng/μl. Sequencing libraries were produced by the use of the Illumina TruSeq stranded-RNA protocol.

### Data acquisition and analysis.

The 24 libraries (3 replicates per condition) underwent 76-bp paired-end sequencing on an Illumina HiSeq 2500 system at the Yale Center for Genomics Analysis (YCGA). Adapter sequences, empty reads, and low-quality sequences were removed. For each read, we trimmed the first six nucleotides and the last nucleotides at the point where the Phred score of an examined base fell below 20 using in-house scripts. Any read that was less than 45 bp in length after trimming was discarded. The remaining trimmed reads were aligned to the N. crassa OR74A v12 genome (6) using Tophat v.2.0.12 with default settings (74). Only reads that mapped to a single unique location within the genome, with ≤2 mismatches in the anchor region of the spliced alignment, were tallied by alignment to exons using HTSeq v0.6.1p1. We also used HiSat2 and StringTie (54, 75, 76) to perform spliced alignments of the reads against the reference genome. Tallies were statistically analyzed with LOX v1.6 (55), yielding relative gene expression levels across the germination time points. For statements involving the statistical significance of multiple genes, *P* values were determined using conservative Bonferroni adjustment (56). We analyzed the differential expression of 800 genes predicted to have isoforms (http://genome.jgi.doe.gov/Neucr2/) using CuffDiff v 2.2.1 (77). We applied the fragment bias correction and strand-specific parameters, leaving other options at the default settings.

### Knockout strains and phenotype identification.

Knockout strains for more than 9,600 genes (43) were acquired from the Fungal Genetic Stock Center (FGSC; 48); those acquired included deletion cassettes for genes in either or both of the two mating types, *mat A* and *mat a*, that regulate mating and sexual development in heterothallic N. crassa (40). Knockout strains of genes that showed a significant (LOX, *P < *0.01) expression difference in the two stage-to-stage expression wild-type strains and a difference in the direction of expression change under the two medium conditions were examined for altered phenotypes during conidial germination. For each investigated strain, 3,000 to 5,000 conidia were plated onto 90-mm diameter plates and monitored. Strains of genotype *mat A* were assayed when available; otherwise, *mat a* strains were used. Genotype *mat a* strains were also assayed in parallel when *mat A* strains exhibited a distinct phenotype. Wild-type strains were monitored alongside each knockout strain on BM and MSM with three replicates under constant white light at 25°C. Germination and growth rates of 20 conidia picked at random were recorded. The knockout strain for NCU08095 that exhibited a significant morphological phenotype was crossed with the wild-type strain, and cosegregation of the observed phenotype with deletion of the gene in the offspring was verified to ensure that the intended deletion was responsible for the mutant phenotype (30, 61, 78).

### Functional enrichment analyses.

The statistical significance of overrepresentation of gene groups in functional categories relative to the whole genome was quantified by calculating *P* values via the hypergeometric distribution using FungiFun (79). To evaluate each functional category, results indicating whether the genes in each functional category were differentially expressed between stages at a higher frequency than expected were assessed in comparison to the genome (FungiFun’s exact *P*) and were based on background gene sets (FungiFun’s adjusted *P*). To achieve significance, we required both an exact *P* value of *<*0.01 and an adjusted *P* value of *<*0.05. Where appropriate, further functional annotation was carried out via the biochemical pathway and annotation data in the Kyoto Encyclopedia of Genes and Genomes (KEGG; 80). Functional annotations were also obtained from FungiDB (81).

### Bayesian network reconstruction.

Biological networks were modeled using the Bayesian network Web server (82) supplied with conidial germination expression data for each culture condition. Input files contained fold changes reflecting differences between adjacent sample points across the experiment [(*X_t_*_+1_ − *X_t_*)/min(*X_t_*, *X_t_*_+1_)]. Global structure learning settings were retained at default settings. The network models depicted are the 50% majority consensuses of 100 models (edge-selection threshold, 0.5; the 100 highest-scoring networks were averaged), calculated without imposition of any structural constraints.
