# Supplementary figures and images for: Metabolism and Development during Conidial Germination in Response to a Carbon-Nitrogen-Rich Synthetic or a Natural Source of Nutrition in Neurospora crassa
Source: mBio. 2019 Mar 26;10(2):e00192-19. doi: 10.1128/mBio.00192-19 (PMC6437048; doi:10.1128/mBio.00192-19)

Fig. S1

BM

MSM

SCM

3 days

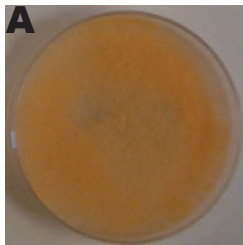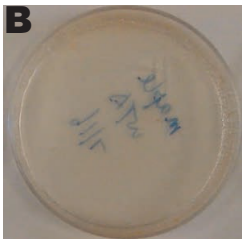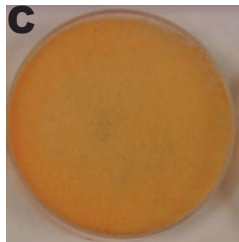

10 days

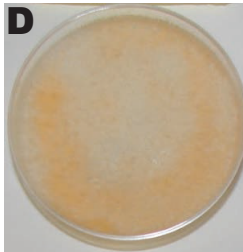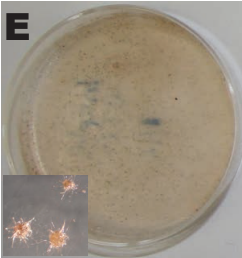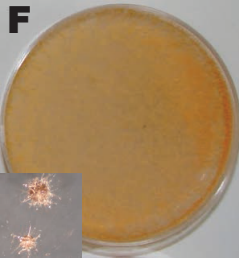

BM

MSM

SCM

Supplement: FIG S1 [file mBio.00192-19-sf001.pdf]

Fig. S2

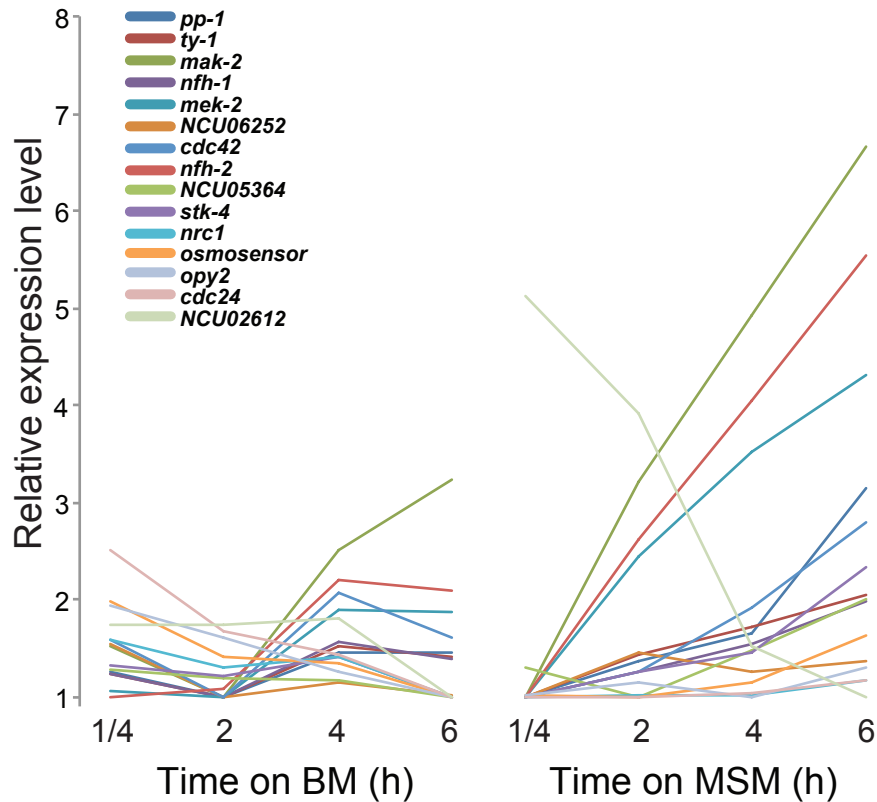

Supplement: FIG S2 [file mBio.00192-19-sf002.pdf]

**Fig. S3**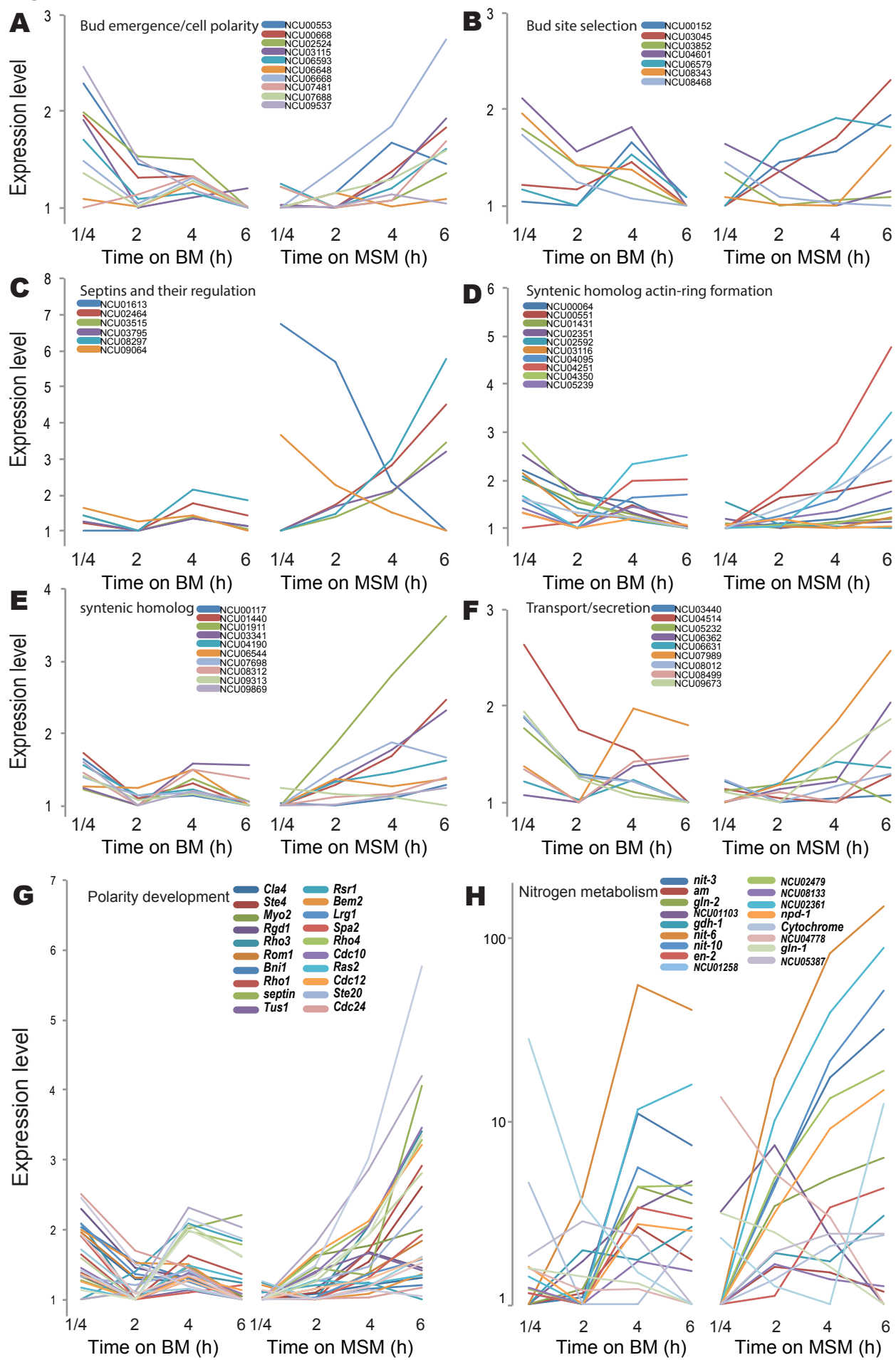

Supplement: FIG S3 [file mBio.00192-19-sf003.pdf]

**Fig. S4**

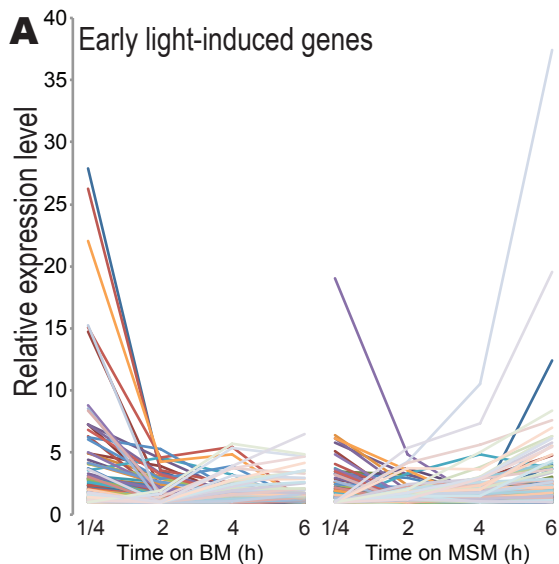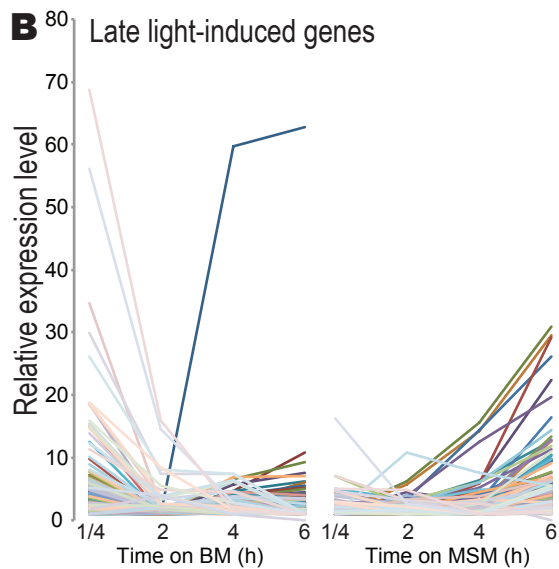

Supplement: FIG S4 [file mBio.00192-19-sf004.pdf]

**Fig. S5**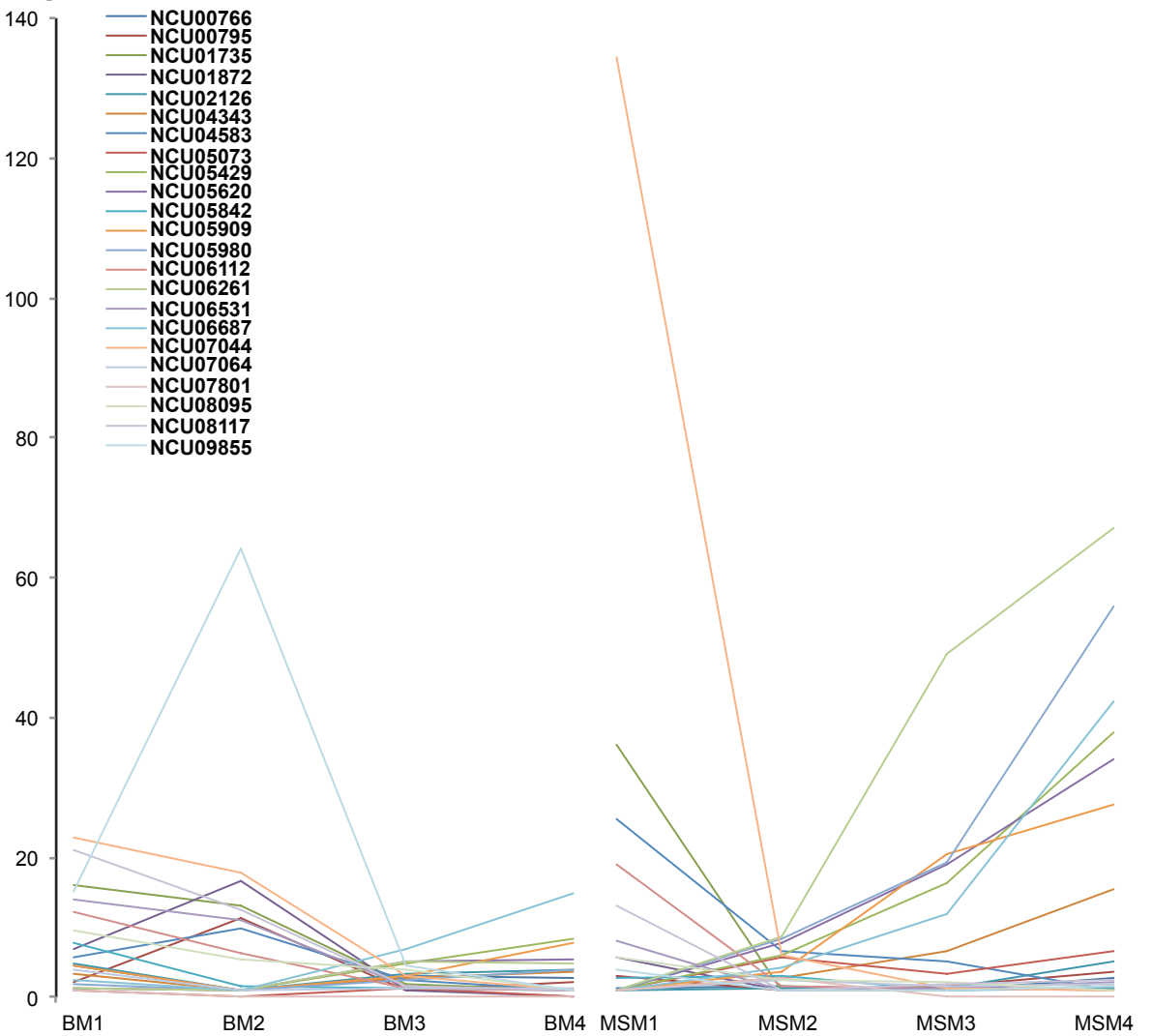

Supplement: FIG S5 [file mBio.00192-19-sf005.pdf]

Fig. S6

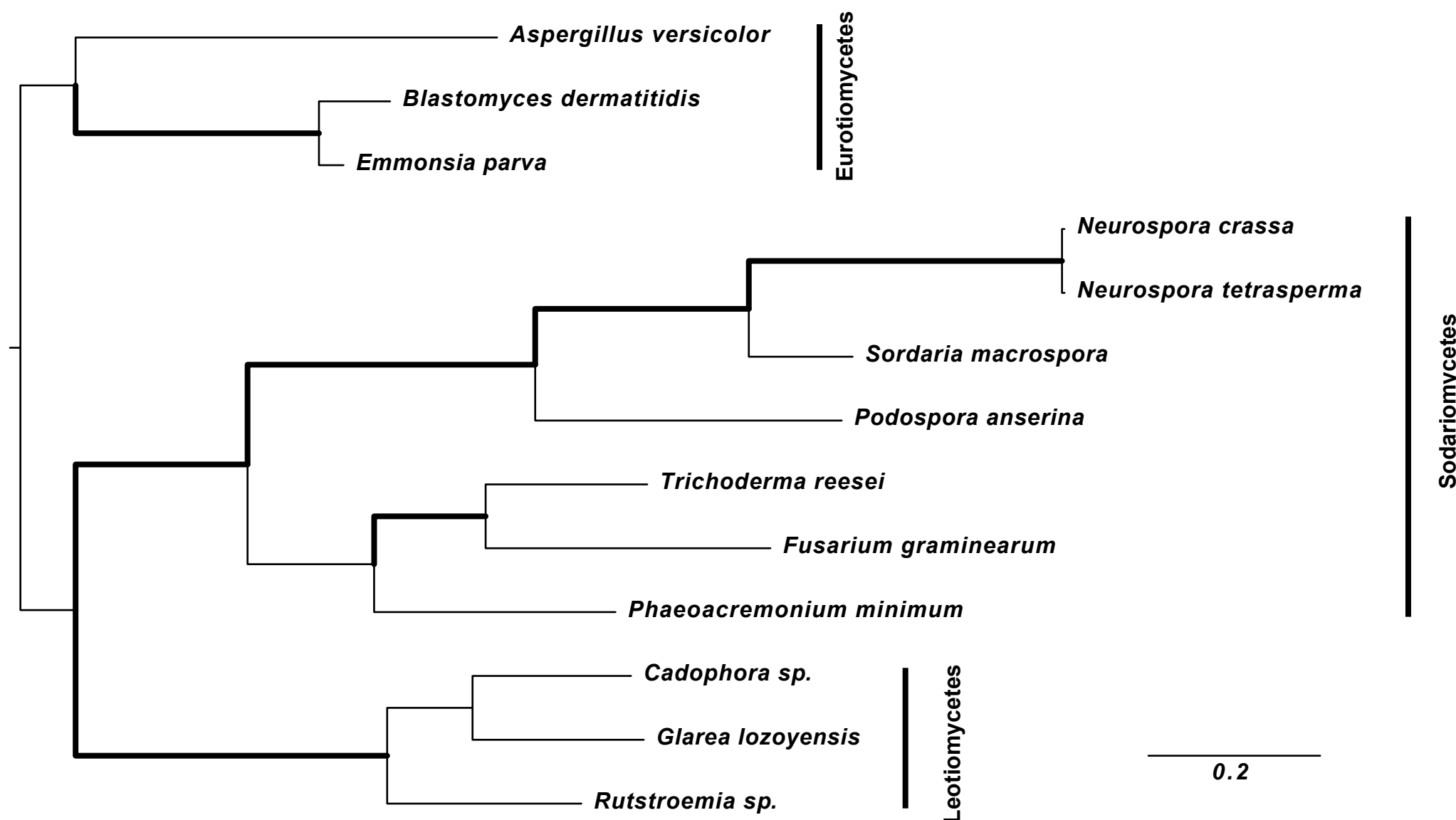

Supplement: FIG S6 [file mBio.00192-19-sf006.pdf]
