# Supplementary material for: Metabolism and Development during Conidial Germination in Response to a Carbon-Nitrogen-Rich Synthetic or a Natural Source of Nutrition in Neurospora crassa
Source: mBio. 2019 Mar 26;10(2):e00192-19. doi: 10.1128/mBio.00192-19 (PMC6437048; doi:10.1128/mBio.00192-19)
Supplement: TABLE S1 [file mBio.00192-19-st001.docx]

**Supplemental table S1. Major components in Bird medium and maple sap medium.**

| **Bird medium (%)** | **Maple sap* medium (%)** |
| --- | --- |
| Glucose **(1.8%)** | Sucrose **(0.88-0.98%)**  Glucose **(0.006-0.077%)**  Fructose **(0.002-0.004%)** |
| NH_4_Cl **(0.134%)** | Minerals (**ppm**)  Boron **0 - 0.04**  Calcium **36 -10684**  Copper **0.01-2.12**  Iron **0.15 - 1.12**  Potassium **62 - 405**  Magnesium **5 - 30.5**  Manganese **0.25 - 23.77**  Sodium **<0.01**  Phosphorous **1.12-30**  Sulphur **0.47 - 12.54**  Zinc **0.08 - 1.2** |
| K_2_HPO_4_  **(0.174%)** |  |
| MES buffer **(0.48%)** |  |
| K_2_SO_4_ **(0.0174%)** |  |
| NaCl **(0.0058%)** |  |
| MgCl_2_^.^6H_2_O **(0.02%)** |  |
| CaCl_2_^.^2H_2_O **(0.007%)** |  |
| Agar **(2%)** |  |
|  | Agar **(2%)** |

*Deirdre Bruce (2003) Production and quality of sap from the big leaf maple (*Acer macrophyllum* Marsh) on Vancouver Island, British Columbia. Mater Dissertation. Department of Geography, University of British Columbia. pp 1-112.
