# Supplementary material for: Metabolism and Development during Conidial Germination in Response to a Carbon-Nitrogen-Rich Synthetic or a Natural Source of Nutrition in Neurospora crassa
Source: mBio. 2019 Mar 26;10(2):e00192-19. doi: 10.1128/mBio.00192-19 (PMC6437048; doi:10.1128/mBio.00192-19)
Supplement: TABLE S3 [file mBio.00192-19-st003.docx]

**Supplemental table S3: Functional analyses for selected genes during conidia germination in *N. crassa*.**

**Supplemental table S3A. Functional groups that were significantly (Adjusted p-value <0.05, FungiFun: https://elbe.hki-jena.de/fungifun/) in the functional enrichment analysis for genes classified with stage-specific expression patterns (Kegg).**

| Stages | Patterns | Genes |  | | KEGG pathway enrichment | | |
| --- | --- | --- | --- | --- | --- | --- | --- |
|  |  |  | pathway ID | pathway name | | Adjusted  P-value | genes / category |
| Stage 1 to 2 | Co-up | 710 | \| 900 \| \| --- \| \| 1110 \| \| 910 \| \| 1200 \| \| 10 \| | \| Terpenoid backbone biosynthesis \| \| --- \| \| Biosynthesis of secondary metabolites \| \| Nitrogen metabolism \| \| Carbon metabolism \| \| Glycolysis / Gluconeogenesis \| | | 0.002317  0.0045717  0.0045717  0.006688  0.028425 | \| 8 / 18 \| \| --- \| \| 38 / 273 \| \| 7 / 17 \| \| 18 / 96 \| \| 9 / 37 \| |
|  | Co-down | 3097 | \| 3008 \| \| --- \| \| 3040 \| \| 3020 \| \| 240 \| \| 230 \| \| 3030 \| \| 3013 \| \| 750 \| \| 770 \| | \| Ribosome biogenesis in eukaryotes \| \| --- \| \| Spliceosome \| \| RNA polymerase \| \| Pyrimidine metabolism \| \| Purine metabolism \| \| DNA replication \| \| RNA transport \| \| Vitamin B6 metabolism \| \| Pantothenate and CoA biosynthesis \| | | 9.3163e-12  7.2599e-9  8.4538e-9  0.000018013  0.000064428  0.00049349  0.00084698  0.038179  0.039856 | \| 54 / 65 \| \| --- \| \| 63 / 88 \| \| 26 / 27 \| \| 44 / 64 \| \| 52 / 82 \| \| 24 / 32 \| \| 52 / 88 \| \| 8 / 9 \| \| 12 / 16 \| |
|  | Up-rgulated in MSM*  down-regulated in BM** | 2434 | \| 3010 \| \| --- \| \| 190 \| \| 4145 \| \| 4146 \| \| 3050 \| \| 563 \| \| 4141 \| | \| Ribosome \| \| --- \| \| Oxidative phosphorylation \| \| Phagosome \| \| Peroxisome \| \| Proteasome \| \| (GPI)-anchor biosynthesis \| \| Protein processing in endoplasmic reticulum \| | | 6.7903e-9  1.7091e-7  0.0011933  0.0021885  0.0056037  0.0067919  0.022972 | \| 72 / 107 \| \| --- \| \| 51 / 72 \| \| 25 / 35 \| \| 28 / 42 \| \| 23 / 34 \| \| 13 / 16 \| \| 38 / 69 \| |
|  | down-regulated in MSM  Up-regulated in BM | 194 | None | None | |  | None |
| Stage 2 to 3 | Co-up | 2627 | \| 3050 \| \| --- \| \| 4141 \| \| 190 \| \| 4145 \| \| 513 \| \| 510 \| \| 100 \| \| 563 \| \| 564 \| \| 4144 \| \| 4146 \| \| 3060 \| \| 4140 \| \| 1100 \| \| 900 \| | \| Proteasome \| \| --- \| \| Protein processing in endoplasmic reticulum \| \| Oxidative phosphorylation \| \| Phagosome \| \| Various types of N-glycan biosynthesis \| \| N-Glycan biosynthesis \| \| Steroid biosynthesis \| \| (GPI)-anchor biosynthesis \| \| Glycerophospholipid metabolism \| \| Endocytosis \| \| Peroxisome \| \| Protein export \| \| Regulation of autophagy \| \| Metabolic pathways \| \| Terpenoid backbone biosynthesis \| | | 8.7372e-13  6.7018e-10  1.2218e-9  1.1704e-7  0.000015128  0.000015767  0.00010581  0.00024013  0.00044242  0.00074768  0.0023183  0.0035907  0.020171  0.024604  0.03806 | \| 33 / 34 \| \| --- \| \| 51 / 69 \| \| 52 / 72 \| \| 29 / 35 \| \| 19 / 22 \| \| 23 / 29 \| \| 15 / 17 \| \| 14 / 16 \| \| 24 / 35 \| \| 27 / 42 \| \| 26 / 42 \| \| 12 / 15 \| \| 11 / 15 \| \| 267 / 693 \| \| 12 / 18 \| |
|  | Co-down | 1191 | \| 1230 \| \| --- \| \| 970 \| \| 400 \| \| 3008 \| \| 1210 \| \| 750 \| \| 3020 \| \| 1110 \| \| 1100 \| \| 260 \| \| 250 \| \| 240 \| \| 770 \| \| 290 \| \| 300 \| \| 380 \| \| 230 \| \| 670 \| \| 450 \| \| 330 \| \| 340 \| | \| Biosynthesis of amino acids \| \| --- \| \| Aminoacyl-tRNA biosynthesis \| \| Phenylalanine, tyrosine, tryptophan biosynthesis \| \| Ribosome biogenesis in eukaryotes \| \| 2-Oxocarboxylic acid metabolism \| \| Vitamin B6 metabolism \| \| RNA polymerase \| \| Biosynthesis of secondary metabolites \| \| Metabolic pathways \| \| Glycine, serine and threonine metabolism \| \| Alanine, aspartate and glutamate metabolism \| \| Pyrimidine metabolism \| \| Pantothenate and CoA biosynthesis \| \| Valine, leucine and isoleucine biosynthesis \| \| Lysine biosynthesis \| \| Tryptophan metabolism \| \| Purine metabolism \| \| One carbon pool by folate \| \| Selenocompound metabolism \| \| Arginine and proline metabolism \| \| Histidine metabolism \| | | 5.0785e-11  1.7777E-06  0.000039745  0.000068798  0.000068798  0.00069338  0.00069338  0.0010324  0.0027632  0.0027722  0.0033857  0.0066121  0.0080289  0.0088488  0.017106  0.018323  0.023494  0.024854  0.028204  0.033773  0.044662 | \| 47 / 109 \| \| --- \| \| 20 / 37 \| \| 13 / 21 \| \| 25 / 65 \| \| 16 / 32 \| \| 7 / 9 \| \| 13 / 27 \| \| 64 / 273 \| \| 133 / 693 \| \| 13 / 31 \| \| 12 / 28 \| \| 20 / 64 \| \| 8 / 16 \| \| 7 / 13 \| \| 6 / 11 \| \| 9 / 22 \| \| 22 / 82 \| \| 6 / 12 \| \| 5 / 9 \| \| 11 / 33 \| \| 5 / 10 \| |
|  | Up-rgulated in MSM  Down-regulated in BM | 497 | \| 600 \| \| --- \| \| 740 \| \| 4011 \| \| 4144 \| \| 480 \| \| 4130 \| | \| Sphingolipid metabolism \| \| --- \| \| Riboflavin metabolism \| \| MAPK signaling pathway - yeast \| \| Endocytosis \| \| Glutathione metabolism \| \| SNARE interactions in vesicular transport \| | | 0.0030969  0.044052  0.044052  0.046991  0.046991  0.046991 | \| 5 / 10 \| \| --- \| \| 4 / 12 \| \| 6 / 29 \| \| 7 / 42 \| \| 5 / 23 \| \| 4 / 15 \| |
|  | Down-regulated in MSM  Up-regulated in BM | 2129 | \| 3010 \| \| --- \| \| 3040 \| \| 3030 \| \| 3013 \| \| 3430 \| \| 3015 \| \| 4113 \| \| 270 \| \| 3440 \| \| 4111 \| | \| Ribosome \| \| --- \| \| Spliceosome \| \| DNA replication \| \| RNA transport \| \| Mismatch repair \| \| mRNA surveillance pathway \| \| Meiosis - yeast \| \| Cysteine and methionine metabolism \| \| Homologous recombination \| \| Cell cycle - yeast \| | | 1.9145e-48  2.8198e-7  2.6383E-06  5.5975E-06  0.00011299  0.0011569  0.010356  0.017163  0.026449  0.035572 | \| 104 / 107 \| \| --- \| \| 54 / 88 \| \| 25 / 32 \| \| 51 / 88 \| \| 18 / 23 \| \| 28 / 47 \| \| 28 / 52 \| \| 21 / 37 \| \| 12 / 18 \| \| 33 / 69 \| |
| Stage 3 to 4 | Co-up | 946 | 3050 | Proteasome | | 4.2567e-24 | 31 / 34 |
|  | Co-down | 2307 | \| 3010 \| \| --- \| \| 3008 \| \| 1230 \| \| 1210 \| \| 970 \| \| 3020 \| \| 300 \| \| 230 \| \| 400 \| \| 290 \| \| 240 \| \| 3013 \| \| 330 \| | \| Ribosome \| \| --- \| \| Ribosome biogenesis in eukaryotes \| \| Biosynthesis of amino acids \| \| 2-Oxocarboxylic acid metabolism \| \| Aminoacyl-tRNA biosynthesis \| \| RNA polymerase \| \| Lysine biosynthesis \| \| Purine metabolism \| \| Phenylalanine, tyrosine, tryptophan biosynthesis \| \| Valine, leucine and isoleucine biosynthesis \| \| Pyrimidine metabolism \| \| RNA transport \| \| Arginine and proline metabolism \| | | 9.2851e-40  2.7298e-11  9.5873e-11  7.1604E-06  0.000028323  0.00023531  0.00023531  0.0028709  0.0029748  0.0056949  0.0095512  0.030412  0.03451 | \| 103 / 107 \| \| --- \| \| 52 / 65 \| \| 75 / 109 \| \| 26 / 32 \| \| 28 / 37 \| \| 21 / 27 \| \| 11 / 11 \| \| 46 / 82 \| \| 16 / 21 \| \| 11 / 13 \| \| 36 / 64 \| \| 45 / 88 \| \| 20 / 33 \| |
|  | Up-rgulated in MSM  Down-regulated in BM | 2657 | \| 4011 \| \| --- \| \| 4120 \| \| 563 \| \| 4113 \| \| 4130 \| | \| MAPK signaling pathway - yeast \| \| --- \| \| Ubiquitin mediated proteolysis \| \| (GPI)-anchor biosynthesis \| \| Meiosis - yeast \| \| SNARE interactions in vesicular transport \| | | 0.00099695  0.00099695  0.014736  0.014736  0.024995 | \| 19 / 29 \| \| --- \| \| 28 / 52 \| \| 11 / 16 \| \| 25 / 52 \| \| 10 / 15 \| |
|  | Down-regulated in MSM  Up-regulated in BM | 193 | \| 1100 \| \| --- \| \| 1200 \| \| 920 \| \| 270 \| \| 190 \| \| 20 \| \| 670 \| \| 620 \| \| 1110 \| \| 1230 \| \| 450 \| | \| Metabolic pathways \| \| --- \| \| Carbon metabolism \| \| Sulfur metabolism \| \| Cysteine and methionine metabolism \| \| Oxidative phosphorylation \| \| Citrate cycle (TCA cycle) \| \| One carbon pool by folate \| \| Pyruvate metabolism \| \| Biosynthesis of secondary metabolites \| \| Biosynthesis of amino acids \| \| Selenocompound metabolism \| | | 5.6539E-06  5.6539E-06  0.00011384  0.0035265  0.0049713  0.010284  0.014171  0.014171  0.020115  0.026257  0.037646 | \| 56 / 693 \| \| --- \| \| 18 / 96 \| \| 7 / 17 \| \| 8 / 37 \| \| 11 / 72 \| \| 6 / 26 \| \| 4 / 12 \| \| 6 / 29 \| \| 23 / 273 \| \| 12 / 109 \| \| 3 / 9 \| |
| Not detected | Maple (312) | 221 | None | None | | None | None |
|  | Bird (336) |  |  |  |  |  |  |

* Cultures on Maple Sap Medium.

** Culture on Bird Medium

**Supplemental table S3B. Functional annotations of genes that were identified for the Bayesian Network of asexual-sexual developmental in responses to environmental factors during the conidial germination.**

| Gene name | Gene ID | Annotated functions in *N. crassa* | References |
| --- | --- | --- | --- |
| ***con-8*** | NCU10997 | asexual reproduction of conidia. | [[1]](https://paperpile.com/c/L5vx25/Rp1r) |
| ***con-13*** | NCU07324 | asexual reproduction of conidia. | [[2]](https://paperpile.com/c/L5vx25/pP9j) |
|  |  |  |  |
| ***cot-1*** | NCU07296 | cell elongation and branching, stress response. | [[3–5]](https://paperpile.com/c/L5vx25/ka8p+iHky+BKpl) |
| ***cot-2*** | NCU04189 | hyphal elongation and branching. | [[6]](https://paperpile.com/c/L5vx25/7r3q) |
| ***cot-3*** | NCU07700 | hyphal elongation and branching. | [[7]](https://paperpile.com/c/L5vx25/tyZ0) |
| ***cot-4*** | NCU03804 | hyphal elongation and branching. | [[6]](https://paperpile.com/c/L5vx25/7r3q) |
| ***cot-5*** | NCU03503 | hyphal elongation and branching. | [[6]](https://paperpile.com/c/L5vx25/7r3q) |
|  |  |  |  |
| ***nit-3*** | NCU05298 | nitrate reductase, nitrogen metabolism. | [[7–9]](https://paperpile.com/c/L5vx25/tyZ0+dybx+HtzB) |
| ***nit-6*** | NCU04720 | nitrate reductase, light-responsive nitrogen metabolism. | [[10–12]](https://paperpile.com/c/L5vx25/9R1l+KfUu+tLWE) |
| ***nit-10*** | NCU07205 | nitrate reductase, nitrogen metabolism. | [[11]](https://paperpile.com/c/L5vx25/KfUu) |
|  |  |  |  |
| ***nop-1*** | NCU10055 | potential green-light sensor, involved in the initiation of perithecia, probably also involved in asexual reproduction of conidia. | [[13–15]](https://paperpile.com/c/L5vx25/iZZe+mhTY+HSZT) |
| ***phy-2*** | NCU05790 | potential red-light sensor, involved in the initiation of perithecia, probably also involved in asexual reproduction of conidia. | [[16–18]](https://paperpile.com/c/L5vx25/bRPb+Gbzq+UV9S) |
|  |  |  |  |
| ***per-1*** | NCU03584 | polyketide synthase, required for female development, involved in melanization of the perithecia. | [[19–21]](https://paperpile.com/c/L5vx25/MLDX+DWhh+etOn) |
| ***pp-1*** | NCU00340 | transcription factor, essential for mating and initiation of perithecia, contribute to vegetative growth. | [[22]](https://paperpile.com/c/L5vx25/EgRx) |
| ***sd*** | NCU07823 | Scytalone dehydratase, involved in melanin synthesis, perithecial pigmentation and seuxal development | [[23]](https://paperpile.com/c/L5vx25/5m6Z) |
| ***tnr-1*** | NCU09390 | terrahydroxynaphthalene reductase-1, involved in melanin synthesis, perithecial pigmentation and seuxal development | [[23]](https://paperpile.com/c/L5vx25/5m6Z) |

1. [Roberts AN, Yanofsky C. Genes expressed during conidiation in Neurospora crassa: characterization of con-8. Nucleic Acids Res. 1989;17: 197–214.](http://paperpile.com/b/L5vx25/Rp1r)

2. [Hager KM, Yanofsky C. Genes expressed during conidiation in Neurospora crassa: molecular characterization of con-13. Gene. 1990;96: 153–159.](http://paperpile.com/b/L5vx25/pP9j)

3. [Ziv C, Kra-Oz G, Gorovits R, März S, Seiler S, Yarden O. Cell elongation and branching are regulated by differential phosphorylation states of the nuclear Dbf2-related kinase COT1 in Neurospora crassa. Mol Microbiol. 2009;74: 974–989.](http://paperpile.com/b/L5vx25/ka8p)

4. [Gorovits R, Yarden O. Environmental suppression of Neurospora crassa cot-1 hyperbranching: a link between COT1 kinase and stress sensing. Eukaryot Cell. 2003;2: 699–707.](http://paperpile.com/b/L5vx25/iHky)

5. [Herold I, Yarden O. Regulation of Neurospora crassa cell wall remodeling via the cot-1 pathway is mediated by gul-1. Curr Genet. 2017;63: 145–159.](http://paperpile.com/b/L5vx25/BKpl)

6. [Resheat-Eini Z, Zelter A, Gorovits R, Read ND, Yarden O. The Neurospora crassa colonial temperature sensitive 2, 4 and 5 (cot-2, cot-4 and cot-5) genes encode regulatory and structural proteins required for hyphal elongation and branching. Fungal Genet Rep. 2008;55: 32–36.](http://paperpile.com/b/L5vx25/7r3q)

7. [Propheta O, Vierula J, Toporowski P, Gorovits R, Yarden O. The Neurospora crassa colonial temperature-sensitive 3 (cot-3) gene encodes protein elongation factor 2. Mol Gen Genet. 2001;264: 894–901.](http://paperpile.com/b/L5vx25/tyZ0)

8. [Tao Y, Marzluf GA. Analysis of a distal cluster of binding elements and other unusual features of the promoter of the highly regulated nit-3 gene of Neurospora crassa. Biochemistry. 1998;37: 11136–11142.](http://paperpile.com/b/L5vx25/dybx)

9. [Chiang TY, Marzluf GA. Binding affinity and functional significance of NIT2 and NIT4 binding sites in the promoter of the highly regulated nit-3 gene, which encodes nitrate reductase in Neurospora crassa. J Bacteriol. 1995;177: 6093–6099.](http://paperpile.com/b/L5vx25/HtzB)

10. [Colandene JD, Garrett RH. Functional dissection and site-directed mutagenesis of the structural gene for NAD(P)H-nitrite reductase in Neurospora crassa. J Biol Chem. 1996;271: 24096–24104.](http://paperpile.com/b/L5vx25/9R1l)

11. [Gao-Rubinelli F, Marzluf GA. Identification and characterization of a nitrate transporter gene in Neurospora crassa. Biochem Genet. 2004;42: 21–34.](http://paperpile.com/b/L5vx25/KfUu)

12. [Fillipovich SI, Bachurina GP, Kritskiĭ MS. [A study of the nitrate and nitrite discharge from the mutant cells of Neurospora crassa lacking nitrate and nitrite reductase activities]. Prikl Biokhim Mikrobiol. 2007;43: 331–337.](http://paperpile.com/b/L5vx25/tLWE)

13. [Bieszke JA, Li L, Borkovich KA. The fungal opsin gene nop-1 is negatively-regulated by a component of the blue light sensing pathway and influences conidiation-specific gene expression in Neurospora crassa. Curr Genet. 2007;52: 149–157.](http://paperpile.com/b/L5vx25/iZZe)

14. [Bieszke JA, Braun EL, Bean LE, Kang S, Natvig DO, Borkovich KA. The nop-1 gene of Neurospora crassa encodes a seven transmembrane helix retinal-binding protein homologous to archaeal rhodopsins. Proc Natl Acad Sci U S A. 1999;96: 8034–8039.](http://paperpile.com/b/L5vx25/mhTY)

15. [Wang Z, Wang J, Li N, Li J, Trail F, Dunlap JC, et al. Light sensing by opsins and fungal ecology: NOP-1 modulates entry into sexual reproduction in response to environmental cues. Mol Ecol. 2018;27: 216–232.](http://paperpile.com/b/L5vx25/HSZT)

16. [Froehlich AC, Noh B, Vierstra RD, Loros J, Dunlap JC. Genetic and molecular analysis of phytochromes from the filamentous fungus Neurospora crassa. Eukaryot Cell. 2005;4: 2140–2152.](http://paperpile.com/b/L5vx25/bRPb)

17. [Olmedo M, Ruger-Herreros C, Luque EM, Corrochano LM. A complex photoreceptor system mediates the regulation by light of the conidiation genes con-10 and con-6 in Neurospora crassa. Fungal Genet Biol. 2010;47: 352–363.](http://paperpile.com/b/L5vx25/Gbzq)

18. [Wang Z, Li N, Li J, Dunlap JC, Trail F, Townsend JP. The Fast-Evolving phy-2 Gene Modulates Sexual Development in Response to Light in the Model Fungus Neurospora crassa. MBio. 2016;7: e02148.](http://paperpile.com/b/L5vx25/UV9S)

19. [Chinnici JL, Fu C, Caccamise LM, Arnold JW, Free SJ. Neurospora crassa female development requires the PACC and other signal transduction pathways, transcription factors, chromatin remodeling, cell-to-cell fusion, and autophagy. PLoS One. 2014;9: e110603.](http://paperpile.com/b/L5vx25/MLDX)

20. [Howe HB Jr, Benson EW. A perithecial color mutant of Neurospora crassa. Mol Gen Genet. 1974;131: 79–83.](http://paperpile.com/b/L5vx25/DWhh)

21. [McCluskey K, Wiest AE, Grigoriev IV, Lipzen A, Martin J, Schackwitz W, et al. Rediscovery by Whole Genome Sequencing: Classical Mutations and Genome Polymorphisms in Neurospora crassa. G3 . 2011;1: 303–316.](http://paperpile.com/b/L5vx25/etOn)

22. [Li D, Bobrowicz P, Wilkinson HH, Ebbole DJ. A mitogen-activated protein kinase pathway essential for mating and contributing to vegetative growth in Neurospora crassa. Genetics. 2005;170: 1091–1104.](http://paperpile.com/b/L5vx25/EgRx)

23. [Ao J, Bandyopadhyay S, Free SJ. Characterization of the Neurospora crassa DHN melanin biosynthetic pathway in developing ascospores and peridium cells. Fungal Biol. 2018; doi:](http://paperpile.com/b/L5vx25/5m6Z)[10.1016/j.funbio.2018.10.005](http://dx.doi.org/10.1016/j.funbio.2018.10.005)
